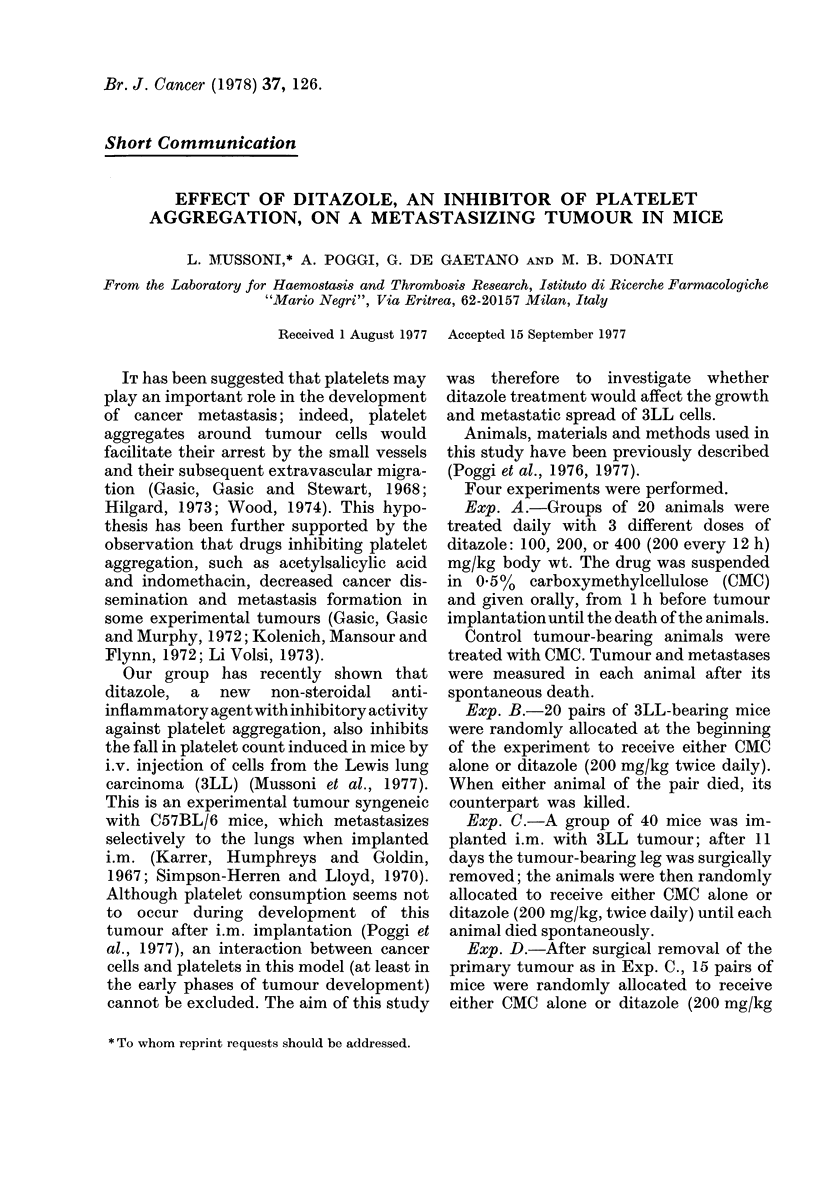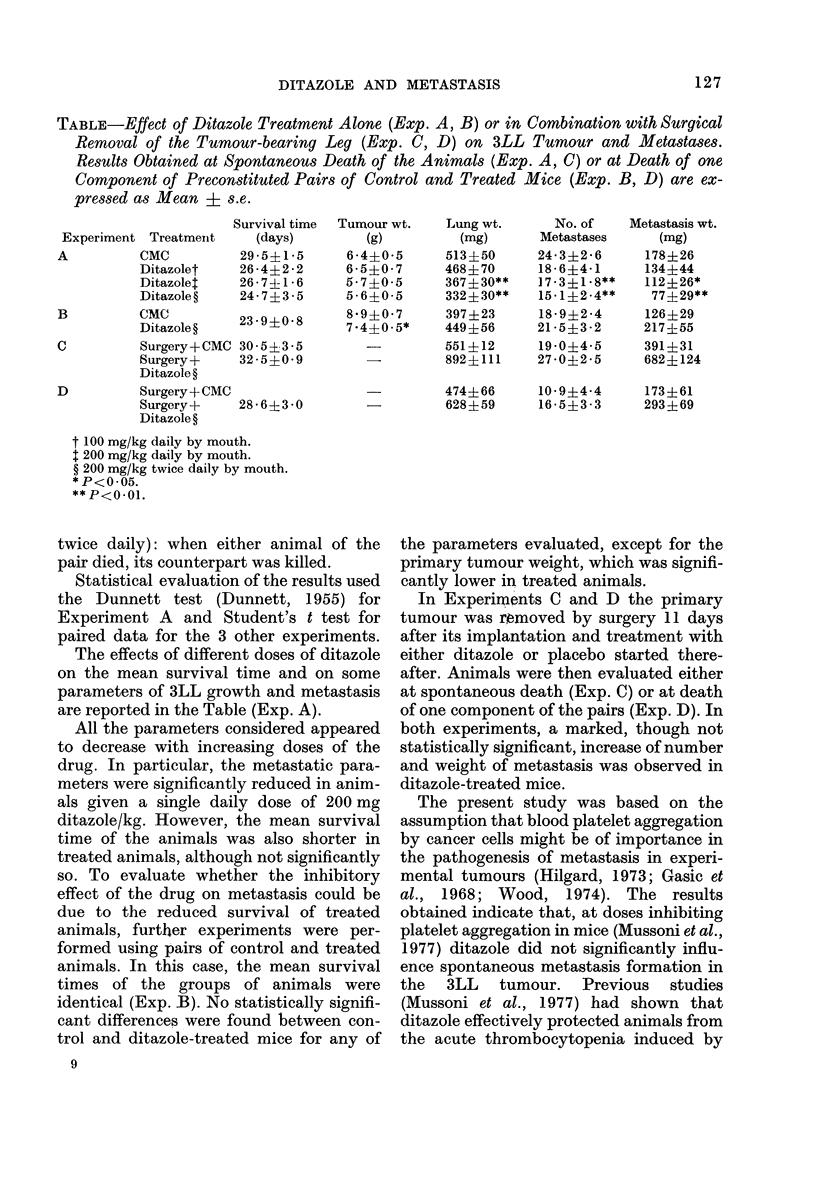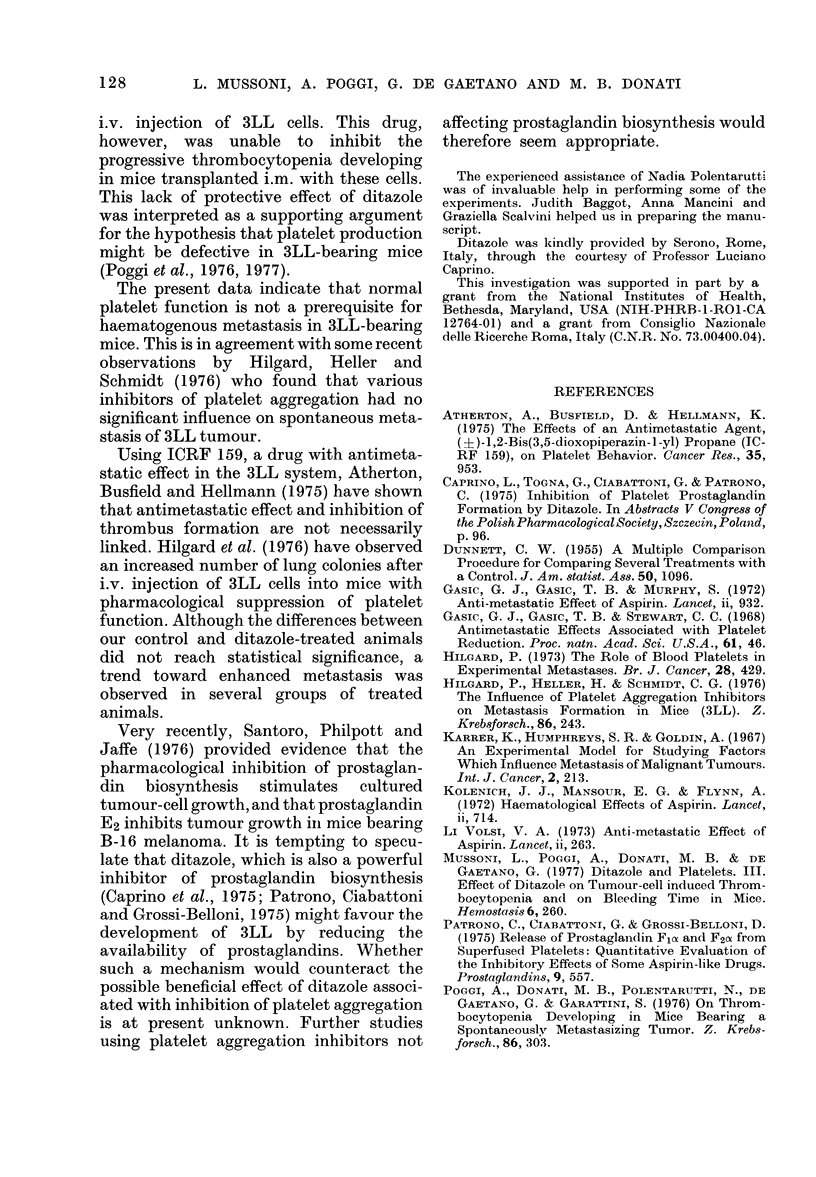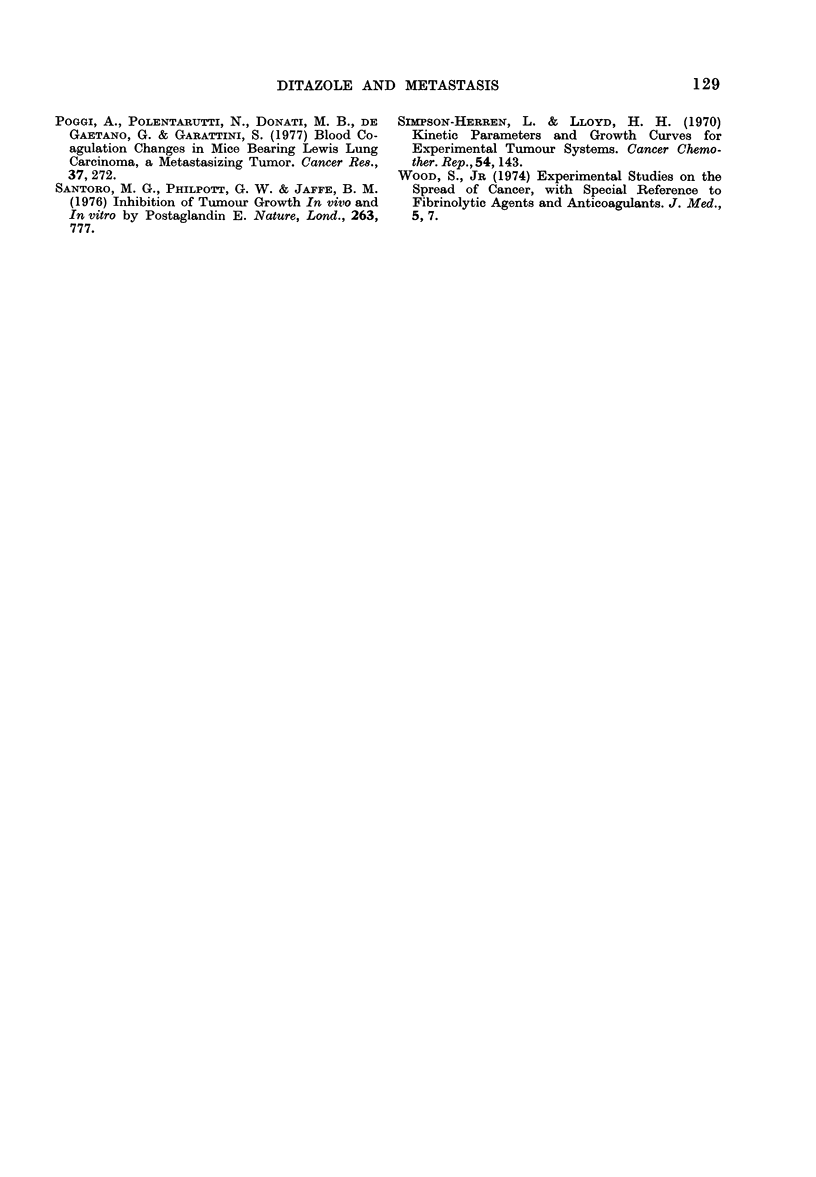# Effect of ditazole, an inhibitor of platelet aggregation, on a metastasizing tumour in mice.

**DOI:** 10.1038/bjc.1978.18

**Published:** 1978-01

**Authors:** L. Mussoni, A. Poggi, G. De Gaetano, M. B. Donati


*Br. J. Cancer* (1978) **37**, 126.

### *Short Communication*

### EFFECT OF DITAZOLE, AN INHIBITOR OF PLATELET AGGREGATION, ON A METASTASIZING TUMOUR IN MICE

L. MUSSONI,* A. POGGI, G. DE GAETANO AND M. B. DONATI

*From the Laboratory for Haemostasis and Thrombosis Research, Istituto di Ricerche Farmacologiche "Mario Negri", Via Eritrea, 62-20157 Milan, Italy*

Received 1 August 1977 Accepted 15 September 1977

It has been suggested that platelets may play an important role in the development of cancer metastasis; indeed, platelet aggregates around tumour cells would facilitate their arrest by the small vessels and their subsequent extravascular migration (Gasic, Gasic and Stewart, 1968; Hilgard, 1973; Wood, 1974). This hypothesis has been further supported by the observation that drugs inhibiting platelet aggregation, such as acetylsalicylic acid and indomethacin, decreased cancer dissemination and metastasis formation in some experimental tumours (Gasic, Gasic and Murphy, 1972; Kolenich, Mansour and Flynn, 1972; Li Volsi, 1973).

Our group has recently shown that ditazole, a new non-steroidal anti-inflammatory agent with inhibitory activity against platelet aggregation, also inhibits the fall in platelet count induced in mice by i.v. injection of cells from the Lewis lung carcinoma (3LL) (Mussoni *et al.*, 1977). This is an experimental tumour syngeneic with C57BL/6 mice, which metastasizes selectively to the lungs when implanted i.m. (Karrer, Humphreys and Goldin, 1967; Simpson-Herren and Lloyd, 1970). Although platelet consumption seems not to occur during development of this tumour after i.m. implantation (Poggi *et al.*, 1977), an interaction between cancer cells and platelets in this model (at least in the early phases of tumour development) cannot be excluded. The aim of this study was therefore to investigate whether ditazole treatment would affect the growth and metastatic spread of 3LL cells.

Animals, materials and methods used in this study have been previously described (Poggi *et al.*, 1976, 1977).

Four experiments were performed.

*Exp. A.*—Groups of 20 animals were treated daily with 3 different doses of ditazole: 100, 200, or 400 (200 every 12 h) mg/kg body wt. The drug was suspended in 0·5% carboxymethylcellulose (CMC) and given orally, from 1 h before tumour implantation until the death of the animals.

Control tumour-bearing animals were treated with CMC. Tumour and metastases were measured in each animal after its spontaneous death.

*Exp. B.*—20 pairs of 3LL-bearing mice were randomly allocated at the beginning of the experiment to receive either CMC alone or ditazole (200 mg/kg twice daily). When either animal of the pair died, its counterpart was killed.

*Exp. C.*—A group of 40 mice was implanted i.m. with 3LL tumour; after 11 days the tumour-bearing leg was surgically removed; the animals were then randomly allocated to receive either CMC alone or ditazole (200 mg/kg, twice daily) until each animal died spontaneously.

*Exp. D.*—After surgical removal of the primary tumour as in Exp. C., 15 pairs of mice were randomly allocated to receive either CMC alone or ditazole (200 mg/kg

* To whom reprint requests should be addressed.

TABLE—*Effect of Ditazole Treatment Alone (Exp. A, B) or in Combination with Surgical Removal of the Tumour-bearing Leg (Exp. C, D) on 3LL Tumour and Metastases. Results Obtained at Spontaneous Death of the Animals (Exp. A, C) or at Death of one Component of Preconstituted Pairs of Control and Treated Mice (Exp. B, D) are expressed as Mean ± s.e.*

| Experiment | Treatment | Survival time (days) | Tumour wt. (g) | Lung wt. (mg) | No. of Metastases | Metastasis wt. (mg) |
|---|---|---|---|---|---|---|
| A | CMC | 29·5±1·5 | 6·4±0·5 | 513±50 | 24·3±2·6 | 178±26 |
| | Ditazole† | 26·4±2·2 | 6·5±0·7 | 468±70 | 18·6±4·1 | 134±44 |
| | Ditazole‡ | 26·7±1·6 | 5·7±0·5 | 367±30** | 17·3±1·8** | 112±26* |
| | Ditazole§ | 24·7±3·5 | 5·6±0·5 | 332±30** | 15·1±2·4** | 77±29** |
| B | CMC | 23·9±0·8 | 8·9±0·7 | 397±23 | 18·9±2·4 | 126±29 |
| | Ditazole§ | | 7·4±0·5* | 449±56 | 21·5±3·2 | 217±55 |
| C | Surgery+CMC | 30·5±3·5 | — | 551±12 | 19·0±4·5 | 391±31 |
| | Surgery+ Ditazole§ | 32·5±0·9 | — | 892±111 | 27·0±2·5 | 682±124 |
| D | Surgery+CMC | 28·6±3·0 | — | 474±66 | 10·9±4·4 | 173±61 |
| | Surgery+ Ditazole§ | | — | 628±59 | 16·5±3·3 | 293±69 |

† 100 mg/kg daily by mouth.

‡ 200 mg/kg daily by mouth.

§ 200 mg/kg twice daily by mouth.

* $P<0·05$.

** $P<0·01$.

twice daily): when either animal of the pair died, its counterpart was killed.

Statistical evaluation of the results used the Dunnett test (Dunnett, 1955) for Experiment A and Student's *t* test for paired data for the 3 other experiments.

The effects of different doses of ditazole on the mean survival time and on some parameters of 3LL growth and metastasis are reported in the Table (Exp. A).

All the parameters considered appeared to decrease with increasing doses of the drug. In particular, the metastatic parameters were significantly reduced in animals given a single daily dose of 200 mg ditazole/kg. However, the mean survival time of the animals was also shorter in treated animals, although not significantly so. To evaluate whether the inhibitory effect of the drug on metastasis could be due to the reduced survival of treated animals, further experiments were performed using pairs of control and treated animals. In this case, the mean survival times of the groups of animals were identical (Exp. B). No statistically significant differences were found between control and ditazole-treated mice for any of the parameters evaluated, except for the primary tumour weight, which was significantly lower in treated animals.

In Experiments C and D the primary tumour was removed by surgery 11 days after its implantation and treatment with either ditazole or placebo started thereafter. Animals were then evaluated either at spontaneous death (Exp. C) or at death of one component of the pairs (Exp. D). In both experiments, a marked, though not statistically significant, increase of number and weight of metastasis was observed in ditazole-treated mice.

The present study was based on the assumption that blood platelet aggregation by cancer cells might be of importance in the pathogenesis of metastasis in experimental tumours (Hilgard, 1973; Gasic *et al.*, 1968; Wood, 1974). The results obtained indicate that, at doses inhibiting platelet aggregation in mice (Mussoni *et al.*, 1977) ditazole did not significantly influence spontaneous metastasis formation in the 3LL tumour. Previous studies (Mussoni *et al.*, 1977) had shown that ditazole effectively protected animals from the acute thrombocytopenia induced by i.v. injection of 3LL cells. This drug, however, was unable to inhibit the progressive thrombocytopenia developing in mice transplanted i.m. with these cells. This lack of protective effect of ditazole was interpreted as a supporting argument for the hypothesis that platelet production might be defective in 3LL-bearing mice (Poggi *et al.*, 1976, 1977).

The present data indicate that normal platelet function is not a prerequisite for haematogenous metastasis in 3LL-bearing mice. This is in agreement with some recent observations by Hilgard, Heller and Schmidt (1976) who found that various inhibitors of platelet aggregation had no significant influence on spontaneous metastasis of 3LL tumour.

Using ICRF 159, a drug with antimetastatic effect in the 3LL system, Atherton, Busfield and Hellmann (1975) have shown that antimetastatic effect and inhibition of thrombus formation are not necessarily linked. Hilgard *et al.* (1976) have observed an increased number of lung colonies after i.v. injection of 3LL cells into mice with pharmacological suppression of platelet function. Although the differences between our control and ditazole-treated animals did not reach statistical significance, a trend toward enhanced metastasis was observed in several groups of treated animals.

Very recently, Santoro, Philpott and Jaffe (1976) provided evidence that the pharmacological inhibition of prostaglandin biosynthesis stimulates cultured tumour-cell growth, and that prostaglandin $E_2$ inhibits tumour growth in mice bearing B-16 melanoma. It is tempting to speculate that ditazole, which is also a powerful inhibitor of prostaglandin biosynthesis (Caprino *et al.*, 1975; Patrono, Ciabattoni and Grossi-Belloni, 1975) might favour the development of 3LL by reducing the availability of prostaglandins. Whether such a mechanism would counteract the possible beneficial effect of ditazole associated with inhibition of platelet aggregation is at present unknown. Further studies using platelet aggregation inhibitors not affecting prostaglandin biosynthesis would therefore seem appropriate.

The experienced assistance of Nadia Polentarutti was of invaluable help in performing some of the experiments. Judith Baggot, Anna Mancini and Graziella Scalvini helped us in preparing the manuscript.

Ditazole was kindly provided by Serono, Rome, Italy, through the courtesy of Professor Luciano Caprino.

This investigation was supported in part by a grant from the National Institutes of Health, Bethesda, Maryland, USA (NIH-PHRB-1-RO1-CA 12764-01) and a grant from Consiglio Nazionale delle Ricerche Roma, Italy (C.N.R. No. 73.00400.04).

### REFERENCES

Atherton, A., Busfield, D. & Hellmann, K. (1975) The Effects of an Antimetastatic Agent, (±)-1,2-Bis(3,5-dioxopiperazin-1-yl) Propane (ICRF 159), on Platelet Behavior. *Cancer Res.*, **35**, 953.

Caprino, L., Togna, G., Ciabattoni, G. & Patrono, C. (1975) Inhibition of Platelet Prostaglandin Formation by Ditazole. In *Abstracts V Congress of the Polish Pharmacological Society, Szczecin, Poland*, p. 96.

Dunnett, C. W. (1955) A Multiple Comparison Procedure for Comparing Several Treatments with a Control. *J. Am. statist. Ass.* **50**, 1096.

Gasic, G. J., Gasic, T. B. & Murphy, S. (1972) Anti-metastatic Effect of Aspirin. *Lancet*, ii, 932.

Gasic, G. J., Gasic, T. B. & Stewart, C. C. (1968) Antimetastatic Effects Associated with Platelet Reduction. *Proc. natn. Acad. Sci. U.S.A.*, **61**, 46.

Hilgard, P. (1973) The Role of Blood Platelets in Experimental Metastases. *Br. J. Cancer*, **28**, 429.

Hilgard, P., Heller, H. & Schmidt, C. G. (1976) The Influence of Platelet Aggregation Inhibitors on Metastasis Formation in Mice (3LL). *Z. Krebsforsch.*, **86**, 243.

Karrer, K., Humphreys, S. R. & Goldin, A. (1967) An Experimental Model for Studying Factors Which Influence Metastasis of Malignant Tumours. *Int. J. Cancer*, **2**, 213.

Kolenich, J. J., Mansour, E. G. & Flynn, A. (1972) Haematological Effects of Aspirin. *Lancet*, ii, 714.

Li Volsi, V. A. (1973) Anti-metastatic Effect of Aspirin. *Lancet*, ii, 263.

Mussoni, L., Poggi, A., Donati, M. B. & de Gaetano, G. (1977) Ditazole and Platelets. III. Effect of Ditazole on Tumour-cell induced Thrombocytopenia and on Bleeding Time in Mice. *Hemostasis* **6**, 260.

Patrono, C., Ciabattoni, G. & Grossi-Belloni, D. (1975) Release of Prostaglandin $F_{1\alpha}$ and $F_{2\alpha}$ from Superfused Platelets: Quantitative Evaluation of the Inhibitory Effects of Some Aspirin-like Drugs. *Prostaglandins*, **9**, 557.

Poggi, A., Donati, M. B., Polentarutti, N., de Gaetano, G. & Garattini, S. (1976) On Thrombocytopenia Developing in Mice Bearing a Spontaneously Metastasizing Tumor. *Z. Krebsforsch.*, **86**, 303.

POGGI, A., POLENTARUTTI, N., DONATI, M. B., DE GAETANO, G. & GARATTINI, S. (1977) Blood Coagulation Changes in Mice Bearing Lewis Lung Carcinoma, a Metastasizing Tumor. *Cancer Res.*, **37**, 272.

SANTORO, M. G., PHILPOTT, G. W. & JAFFE, B. M. (1976) Inhibition of Tumour Growth *In vivo* and *In vitro* by Postaglandin E. *Nature, Lond.*, **263**, 777.

SIMPSON-HERREN, L. & LLOYD, H. H. (1970) Kinetic Parameters and Growth Curves for Experimental Tumour Systems. *Cancer Chemother. Rep.*, **54**, 143.

WOOD, S., JR (1974) Experimental Studies on the Spread of Cancer, with Special Reference to Fibrinolytic Agents and Anticoagulants. *J. Med.*, **5**, 7.